# Supplementary material for: Targeted Delivery of Gene Silencing in Fungi Using Genetically Engineered Bacteria
Source: J Fungi (Basel). 2021 Feb 9;7(2):125. doi: 10.3390/jof7020125 (PMC7914413; doi:10.3390/jof7020125)
Supplement: Supplementary file 1 [file jof-07-00125-s001.pdf]

Supplementary Materials:

Targeted delivery of gene silencing in fungi using genetically engineered bacteria.

Jonathan Niño-Sánchez[1,2], Li-Hung Chen[1,3], Jorge Teodoro De Souza[1,5], Sandra Mosquera[1,4], Ioannis Stergiopoulos[1,*]

The following supplementary materials are available for the article:

Figure S1: Circular maps of genetically engineered plasmids used for the production of dsRNA in *E. coli* HT115(DE3).

Figure S2: Bacterial growth curves of the *E. coli* HT115(DE3) strains transformed with the plasmid vectors used to generate dsRNAs against *AflC* (HT115/*AflC*$_{Con}$), *BcSAS1* (HT115/*BcSAS1*$_{Ind}$), or *eGFP* (HT115/*eGFP*$_{Con}$ and HT115/*eGFP*$_{Ind}$).

Figure S3: Effect of bacterial concentration on RNAi-induced silencing.

Figure S4: Effect of contact time between fungi and bacteria on RNAi-induced silencing.

Table S1: Primers used in this study.

Table S2: Expression levels of *AflC* from *A. flavus* and *BcSAS1* from *B. cinerea* in *in vitro* cultures of the fungi with living or lysed *E. coli* HT115(DE3) bacteria producing dsRNAs against *AflC* (HT115/*AflC*$_{Con}$) or *BcSAS1* (HT115/*BcSAS1*$_{Ind}$), respectively.

Table S3: Differences in expression levels of *AflC* from *A. flavus* among *in vitro* cultures of the fungus with living or lysed *E. coli* HT115(DE3) bacteria producing dsRNAs against *eGFP*.

Table S4: Differences in expression levels of *BcSAS1* from *B. cinerea* among *in vitro* cultures of the fungus with living or lysed *E. coli* HT115(DE3) bacteria producing dsRNAs against *eGFP*.

Table S5: Expression levels of AflC from *A. flavus* and BcSAS1 from *B. cinerea* over time in *in vitro* cultures of the two fungi with living or lysed *E. coli* HT115(DE3) bacteria producing dsRNAs against *AflC* (HT115/AflCCon) or *BcSAS1* (HT115/BcSAS1Ind), respectively.

Table S6: Differences in the expression levels of *AflC* from *A. flavus* over time among *in vitro* cultures of the fungus with living or lysed *E. coli* HT115(DE3) bacteria producing dsRNAs against *eGFP*.

Table S7: Amount of aflatoxins produced by *A. flavus* over time in *in vitro* cultures of the fungus with living or lysed *E. coli* HT115(DE3) bacteria producing dsRNAs against *AflC* or *eGFP*.

Table S8: Differences in the expression levels of *BcSAS1* from *B. cinerea* over time among *in vitro* cultures of the fungus with living or lysed *E. coli* HT115(DE3) bacteria producing dsRNAs against *eGFP*.

Table S9: Amount of fungal biomass generated by B. cinerea over time in *in vitro* cultures of the fungus with living or lysed *E. coli* HT115(DE3) bacteria producing dsRNAs against *BcSAS1* or *GFP*.

Table S10: Expression levels of *AflC* from *A. flavus* and the amount of aflatoxins produced by the fungus on maize kernels, following its exposure to living or lysed *E. coli* HT115(DE3) bacteria producing dsRNAs against *AflC* or *eGFP*.

Table S11: Expression levels of *BcSAS1* from *B. cinerea* and size of infection areas produced by the fungus on leaves of *N. benthamiana*, following its exposure to living or lysed *E. coli* HT115(DE3) bacteria producing dsRNAs against *BcSAS1* or *eGFP*.

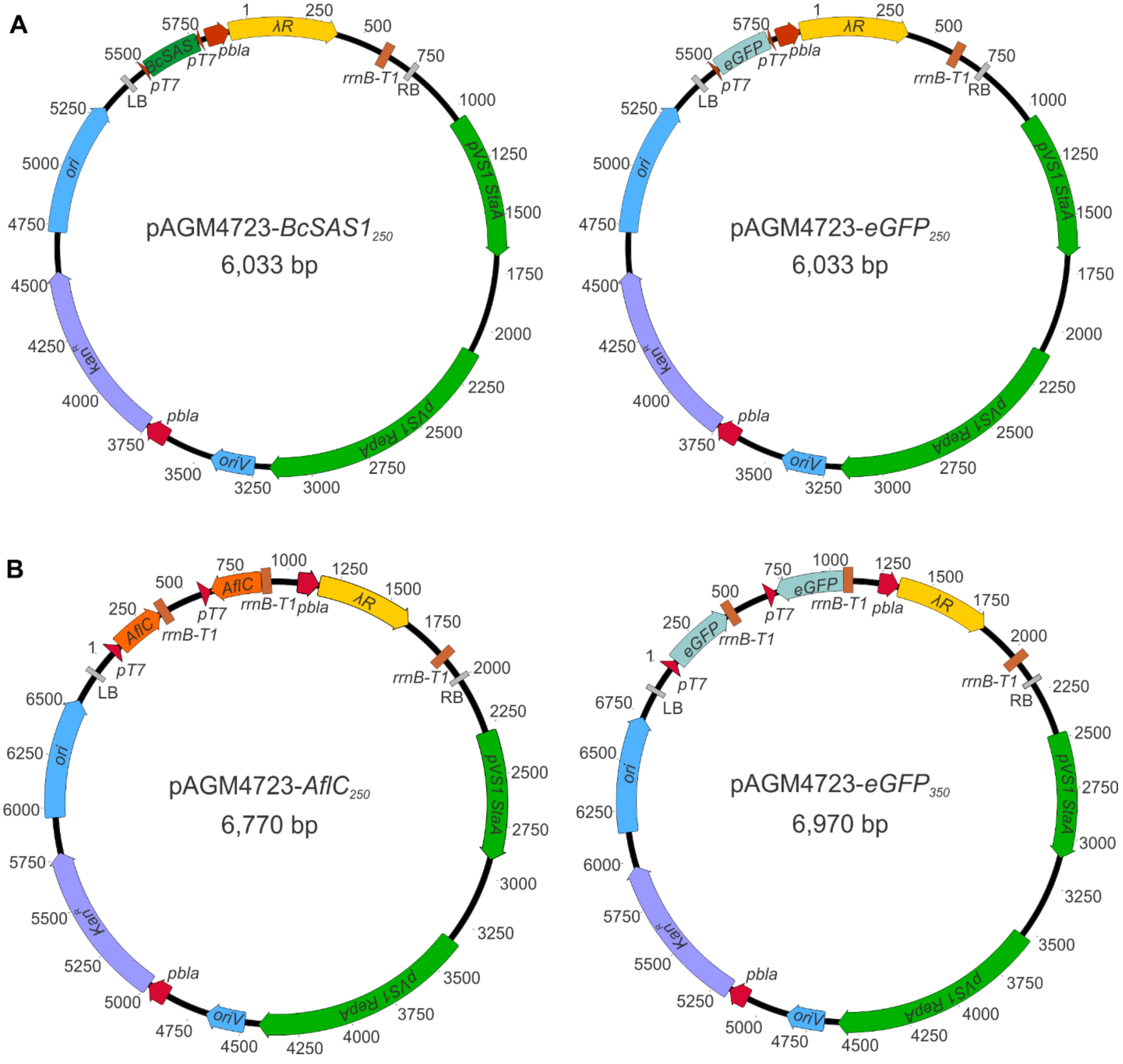

Figure S1. Circular maps of genetically engineered plasmids used for the production of dsRNA in *Escherichia coli* HT115(DE3).

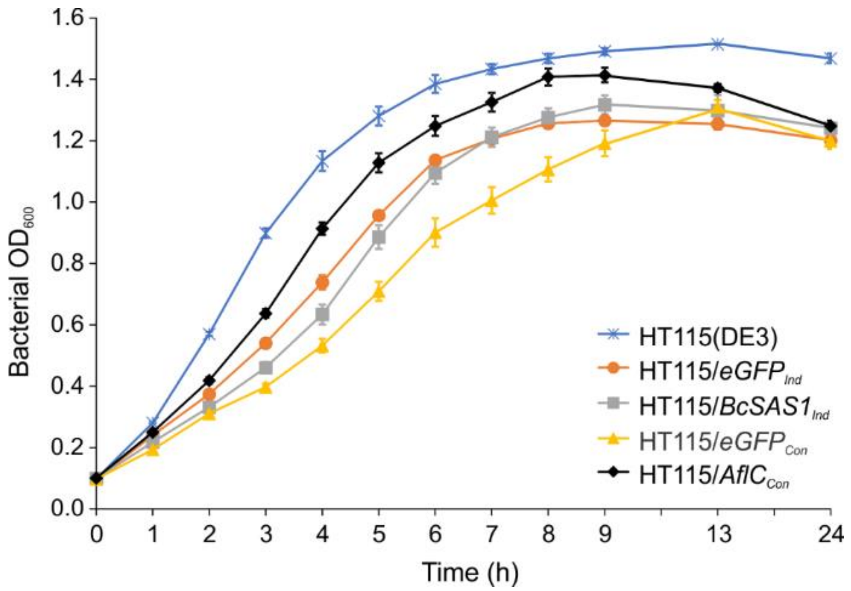

Figure S2. Bacterial growth curves of the *Escherichia coli* HT115(DE3) strains transformed with the plasmid vectors used to generate dsRNAs against *AflC* (HT115/*AflC*$_{Con}$), *BcSAS1* (HT115/*BcSAS1*$_{Ind}$), or eGFP (HT115/*eGFP*$_{Con}$ and HT115/*eGFP*$_{Ind}$). The growth curve of the untransformed *E. coli* HT115(DE3) strain in also shown for comparison. Bacteria were grown at 37°C in LB liquid medium supplemented with tetracycline and kanamycin. Kanamycin was not added to the HT155(DE3) culture, whereas strains HT115/*BcSAS1*$_{Ind}$ and HT115/*eGFP*$_{Ind}$ were induced with 1.0 mM IPTG when they reached an OD_{600} of approximately 0.8. The growth curves show that the transformed HT115(DE3) strains grow at slightly different rates, depending on the dsRNAs that they are producing. Error bars depict standard deviation (SD) obtained from 3 measured point, with each point representing a different bacterial culture (i.e. not technical replicates of the same culture).

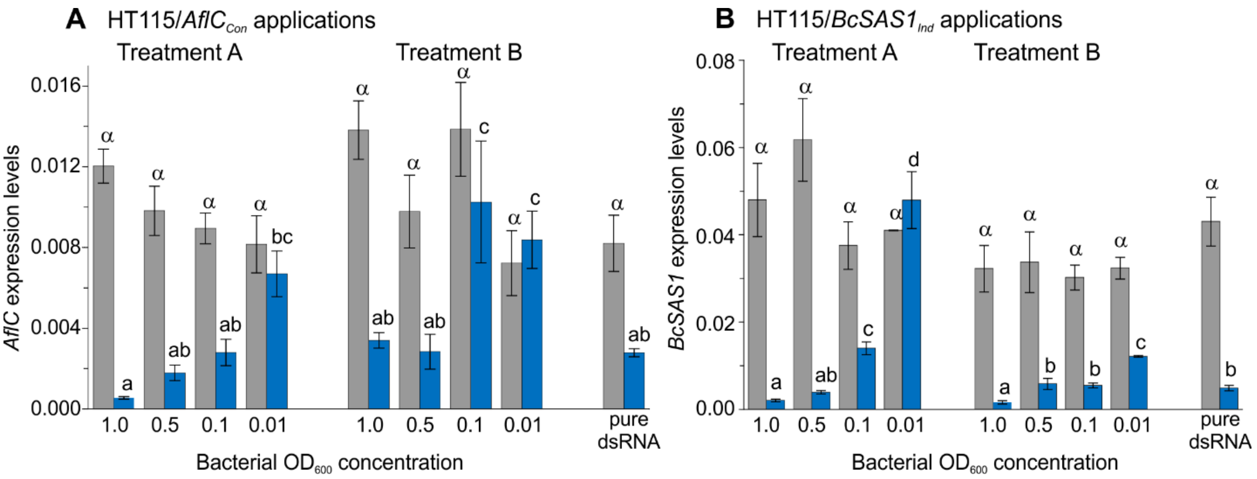

Figure S3. Effect of bacterial concentration on RNAi-induced silencing. (A-B) Expression levels of *AflC* from *Aspergillus flavus* (Panel A) and *BcSAS1* from *Botrytis cinerea* (Panel B), in *in vitro* co-cultures of the two fungi with living (Treatment A) or lysed (Treatment B) *Escherichia coli* HT115(DE3) bacteria producing dsRNAs against *AflC* (HT115/$AflC_{Con}$) or *BcSAS1* (HT115/$BcSAS1_{Ind}$), respectively (test applications). The expression levels of *AflC* and *BcSAS1* are shown relative to their expression when the two fungi are co-incubated with control HT115(DE3) bacteria producing dsRNAs against *eGFP* (HT115/$eGFP_{Con}$ and HT115/$eGFP_{Ind}$, respectively; control applications). Living bacteria or their whole-cell autolysates, obtained from the OD_{600} 1.0, 0.5, 0.1, and 0.01 cultures, were added to the fungal cultures, and the expression levels of *AflC* or *BcSAS*1 were measured at 12h of co-incubation. For comparison, *AflC* and *BcSAS1* silencing levels achieved by the addition in the *in vitro* cultures of *A. flavus* or *B. cinerea* of 15 μg (12.5 nM) of purified dsRNAs against each gene, respectively were also evaluated. Significant differences in the expression levels of *AflC* and *BcSAS1* among the control applications (grey bars) or among the test applications (blue bars) were tested by a one-way ANOVA followed by a Tukey's HSD test and significantly different groups ($P < 0.05$) are indicated by Greek (control applications) or Roman (test applications) letters above the bars. Error bars in the figure indicate standard deviations (SD) obtained from three biological replicates.

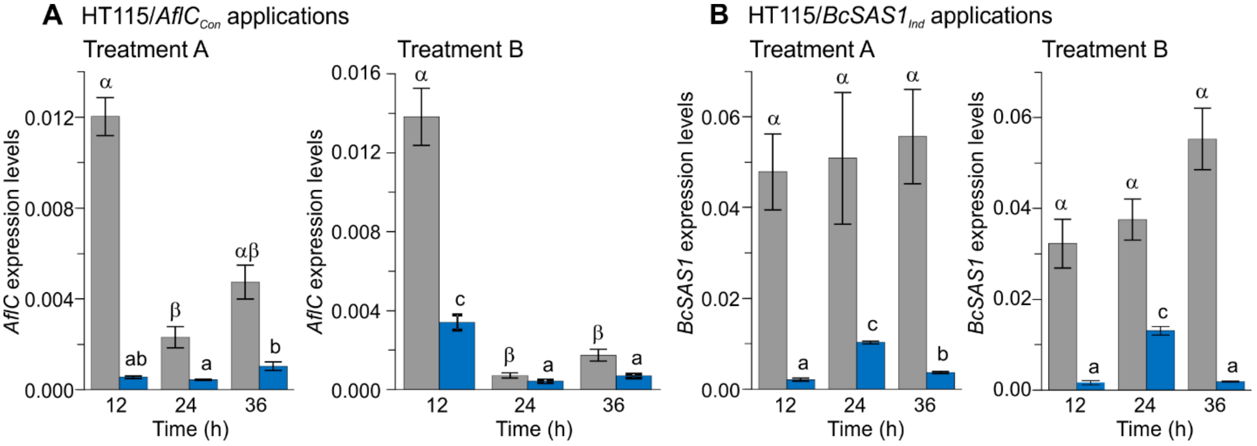

Figure S4. Effect of contact time between fungi and bacteria on RNAi-induced silencing. (A-B) Expression levels over time of *AflC* from *Aspergillus flavus* (Panel A) and *BcSAS1* from *Botrytis cinerea* (Panel B), in *in vitro* co-cultures of the two fungi with living (Treatment A) or lysed (Treatment B) *Escherichia coli* HT115(DE3) bacteria producing dsRNAs against *AflC* (HT115/$AflC_{Con}$) or *BcSAS1* (HT115/$BcSAS1_{Ind}$), respectively (test applications). The expression levels of *AflC* and *BcSAS1* are shown relative to their expression when the two fungi are co-incubated with control HT115(DE3) bacteria producing dsRNAs against *eGFP* (HT115/$eGFP_{Con}$ and HT115/$eGFP_{Ind}$; control applications). Living bacteria or their whole-cell autolysates, obtained from the OD_{600} 1.0 culture, were added to the fungal cultures and the expression levels of *AflC* or *BcSAS*1 were measured at 12h, 24h, and 36h of co-incubation. Significant differences in the expression levels of *AflC* and *BcSAS1* among the control applications (grey bars) or among the test applications (blue bars) were tested by a one-way ANOVA followed by a Tukey's HSD test, and significantly different groups (P< 0.05) are indicated by Greek (control applications) or Roman (test applications) letters above the bars. Error bars in the figure indicate standard deviations (SD) obtained from three biological replicates.

S1 Table. Primers used in this study.

Primer name	Primer sequence (5′ - 3′)	Description and function
AflC-F<br>AflC-R	GTGAAGACGTGCAAGCCCAGAACATTGACACATACT<br>GTGAAGACGTTTATTGTCCAATCCTGTGTGACCA	*AflC* RNAi fragment for pAGM4723-*AflC*$_{250}$
AflCr-F<br>AflCr-R	GTGAAGACGTTTATGCCCAGAACATTGACACATACT<br>GTGAAGACGTGCAATGTCCAATCCTGTGTGACCA	*AflC* RNAi rev-fragment for pAGM4723-*AflC*$_{250}$
PpntII-F:<br>PpntII-R:	GAAGACATGGAGACGCAAGCGCAAAGAGAAAG<br>GAAGACATCATTGAACCTGCGTGCAATCCATC	*PKanR* promoter for RNAi expression in pAGM4723-*AflC*$_{250}$ and pAGM4723-*AflC*$_{250}$
rrnB_T1-term-F<br>rrnB_T1-term-R	GTGAAGACGTATAAAACGAAAGGCTCAGTCGA<br>GTGAAGACGTAGCGTTCCATGTCGGCAGAATGCT	*rrnB* terminator for RNAi expression in pAGM4723-*AflC*$_{250}$ and pAGM4723-*AflC*$_{250}$
EndoR_F<br>EndoR_R	TTGAAGACAAACTACGCGGAACCCCTATTTGT<br>TTGAAGACAAGTAAATTTGTCCTACTCAGGAGAG	Endolysin *R* gene cassette for pAGM4723
EGFP 342 F<br>EGFP342-R	GTGAAGACGTGCAAGACGTAAACGGCCACAAGTT<br>GTGAAGACGTTTATTTGCCGTCCTCCTTGAAGTC	*eGFP* RNAi fragment for pAGM4723-*eGFP*$_{350}$
EGFP342-F-reversed<br>EGFP342-R-reversed	GTGAAGACGTTTATGACGTAAACGGCCACAAGTT<br>GTGAAGACGTGCAATTGCCGTCCTCCTTGAAGTC	*eGFP* RNAi rev-fragment for pAGM4723-*eGFP*$_{350}$
BSAS1_T7_F<br>BcSAS1_T7_R	CCACAGAAGACGATGCCTAATACGACTCACTATAGGACAAACGAGTTGTATCCAC<br>CCACAGAAGACGATTGCTAATACGACTCACTATAGACAACACAACATGCCTC	*BcSAS1* RNAi fragment for pAGM4723-*BcSAS1*$_{250}$
G250_T7_F<br>G250_T7_R	ACAGAAGACGATGCCTAATACGACTCACTATAGCGAGGGCGAGGGCGATGCCA<br>ACAGAAGACGATTGCTAATACGACTCACTATAGGAACTTCACCTCGGCGCGG	*eGFP* RNAi fragment for pAGM4723-*eGFP*$_{250}$
qGADPH F<br>qGADPH R	AGGAATACAAGACCGACATC<br>CTCAACGAGACCGAAGTTGT	RT-qPCR gene expression
qAflC F<br>qAflC R	GCGACTAGACTTGGTTGAGACTGG<br>CGGTGTCAGTCCTTGTCTCTGTAC	RT-qPCR gene expression

qBcActin F	CAAGATCATTGCACCACCCGAGAG	RT-qPCR gene expression
qBcActin R	GTCCGGACTCGTCGTACTCTTGC	
qBcSAS1 F	GGGATACGGCGGGTCAGGAG	RT-qPCR gene expression
qBcSAS1 R	GACGTTGCTGAACCAGGTGCGG	

Table S2. Expression levels of *AflC* from *Aspergillus flavus* and *BcSAS1* from *Botrytis cinerea* in *in vitro* cultures of the fungi with living (Treatment A) or lysed (Treatment B) *Escherichia coli* HT115(DE3) bacteria producing dsRNAs against *AflC* (HT115/*AflC*$_{Con}$) or *BcSAS1* (HT115/*BcSAS1*$_{Ind}$), respectively. The expression levels of *AflC* and *BcSAS1* are shown relative to their expression when the two fungi are co-incubated with control HT115(DE3) bacteria producing dsRNAs against *eGFP* (HT115/*eGFP*$_{Con}$ and HT115/*eGFP*$_{Ind}$, respectively). Living bacteria or their whole-cell autolysates, obtained from the OD_{600} 1.0, 0.5, 0.1, and 0.01 cultures, were added to the fungal cultures and the expression levels of *AflC* or *BcSAS*1 were measured at 12h of co-incubation. Standard deviations (SD; ±) were obtained from three biological replicates. Significant differences between control and test applications were determined by a one-way ANOVA followed by a Tukey's HSD test, and *P* values are reported.

Treatment - Target gene	OD_{600} 1.0	OD_{600} 0.5	OD_{600} 0.1	OD_{600} 0.01
**Treatment A – *AflC***	0.046 ± 0.005	0.183 ± 0.156	0.313 ± 0.168	0.820 ± 0.215
(i.e. dsRNAs against *AflC*)	*P*=0.003 **	*P*=0.032 *	*P*=0.041*	*P*=0.638
**Treatment A - *BcSAS1***	0.043 ± 0.019	0.063 ± 0.016	0.373 ± 0.164	1.169 ± 0.238
(i.e. dsRNAs against *BcSAS1*)	*P*=0.001 **	*P*=0.002 **	*P*=0.048 *	*P*=0.854
**Treatment B – *AflC***	0.246 ± 0.163	0.294 ± 0.188	0.743 ± 0.200	1.160 ± 0.174
(i.e. dsRNAs against *AflC*)	*P*=0.002 **	*P*=0.022 *	*P*=0.562	*P*=0.900
**Treatment B – *BcSAS1***	0.050 ± 0.045	0.173 ± 0.089	0.181 ± 0.075	0.375 ± 0.058
(i.e. dsRNAs against *BcSAS1*)	*P*=0.004 **	*P*=0.031 *	*P*=0.005 **	*P*=0.004 **

* represents a significant difference at $P < 0.05$; ** represents a significant difference at $P < 0.01$.

Table S3. Differences in expression levels of *AflC* from *Aspergillus flavus* among *in vitro* cultures of the fungus with living (Treatment A) or lysed (Treatment B) *Escherichia coli* HT115(DE3) bacteria producing dsRNAs against *eGFP* (HT115/$eGFP_{Con}$; control applications). Living bacteria, or their whole-cell autolysates, obtained from the OD_{600} 1.0, 0.5, 0.1, 0.01, and 0 (axenic) cultures, were added to the fungal cultures and the expression of *AflC* was measured at 12h of co-incubation. Values in bold in the diagonal cells of the table represent the absolute expression of *AflC* in the co-cultures of the fungus with the living bacteria or their whole-cell autolysates. Standard deviations (SD; ±) were obtained from three biological replicates. Values in cells in the lower triangular part of the table indicate the fold-change of the expression levels of *AflC* in the rows as compared to its expression levels in the matching column. Significant differences in the expression levels of *AflC* between rows and columns were determined by a one-way ANOVA followed by a Tukey's HSD test, and *P* values are reported.

	**Treatment A OD_{600} 1.0**	**Treatment A OD_{600} 0.5**	**Treatment A OD_{600} 0.1**	**Treatment A OD_{600} 0.01**	**Treatment B OD_{600} 1.0**	**Treatment B OD_{600} 0.5**	**Treatment B OD_{600} 0.1**	**Treatment B OD_{600} 0.01**	**Axenic culture**
**Treatment A OD_{600} 1.0**	**0.01203 ± 0.00084**								
**Treatment A OD_{600} 0.5**	1.23 *P*=1.000	**0.00982 ± 0.00122**							
**Treatment A OD_{600} 0.1**	1.34 *P*=0.902	1.10 *P*=0.975	**0.00894 ± 0.00076**						
**Treatment A OD_{600} 0.01**	1.47 *P*=0.691	1.20 *P*=0.852	1.10 *P*=1.000	**0.00816 ± 0.00141**					
**Treatment B OD_{600} 1.0**	0.87 *P*=0.999	0.71 *P*=0.984	0.65 *P*=0.553	0.59 *P*=0.317	**0.01382 ± 0.00144**				
**Treatment B OD_{600} 0.5**	1.23 *P*=0.988	1.00 *P*=0.999	0.91 *P*=1.000	0.83 *P*=0.993	1.41 *P*=0.795	**0.00979 ± 0.00181**			
**Treatment B OD_{600} 0.1**	0.87 *P*=0.999	0.71 *P*=0,983	0.65 *P*=0.544	0.59 *P*=0.311	1.00 *P*=1.000	0.71 *P*=0.787	**0.01386 ± 0.00233**		
**Treatment B OD_{600} 0.01**	1.23 *P*=0.370	1.36 *P*=0.542	1.24 *P*=0.982	1.13 *P*=1.000	1.91 *P*=0.128	1.35 *P*=0.881	1.92 *P*=0.125	**0.00723 ± 0.00160**	
**Axenic culture**	1.47 *P*=0.709	1.20 *P*=0.865	1.09 *P*=1.000	0.99 *P*=1.000	1.68 *P*=0.332	1.19 *P*=0.995	1.69 *P*=0.325	0.88 *P*=0.995	**0.00820 ± 0.00139**

Table S4. Differences in expression levels of *BcSAS1* from *Botrytis cinerea* among *in vitro* cultures of the fungus with living (Treatment A) or lysed (Treatment B) *Escherichia coli* HT115(DE3) bacteria producing dsRNAs against *eGFP* (HT115/$eGFP_{Ind}$; control applications). Living bacteria, or their whole-cell autolysates, obtained from the OD_{600} 1.0, 0.5, 0.1, 0.01, and 0 (axenic) cultures, were added to the fungal cultures and the expression of *BcSAS1* was measured at 12h of co-incubation. Values in bold in the diagonal cells of the table represent the absolute expression of *BcSAS1* in the co-cultures of the fungus with the living bacteria or their whole-cell autolysates. Standard deviations (SD; ±) were obtained from three biological replicates. Values in cells in the lower triangular part of the table indicate the fold-change of the expression levels of *BcSAS1* in the rows as compared to its expression levels in the matching column. Significant differences in the expression levels of *BcSAS1* between rows and columns were determined by a one-way ANOVA followed by a Tukey's HSD test, and *P* values are reported.

	**Treatment A OD_{600} 1.0**	**Treatment A OD_{600} 0.5**	**Treatment A OD_{600} 0.1**	**Treatment A OD_{600} 0.01**	**Treatment B OD_{600} 1.0**	**Treatment B OD_{600} 0.5**	**Treatment B OD_{600} 0.1**	**Treatment B OD_{600} 0.01**	**Axenic culture**
**Treatment A OD_{600} 1.0**	**0.04799 ± 0.00838**								
**Treatment A OD_{600} 0.5**	0.78 *P*=0.929	**0.06174 ± 0.00944**							
**Treatment A OD_{600} 0.1**	1.28 *P*=0.937	1.64 *P*=0.293	**0.03755 ± 0.00543**						
**Treatment A OD_{600} 0.01**	1.17 *P*=0.996	1.51 *P*=0.528	0.92 *P*=1.000	**0.04102 ± 0.00008**					
**Treatment B OD_{600} 1.0**	1.49 *P*=0.561	1.91 *P*=0.079 °	1.16 *P*=0.997	1.27 *P*=0.944	**0.03224 ± 0.00535**				
**Treatment B OD_{600} 0.5**	1.42 *P*=0.697	1.83 *P*=0.120	1.11 *P*=1.000	1.22 *P*=0.983	0.95 *P*=1.000	**0.03373 ± 0.00694**			
**Treatment B OD_{600} 0.1**	1.59 *P*=0.377	2.04 *P*=0.069 °	1.24 *P*=0.968	1.36 *P*=0.825	1.07 *P*=1.000	1.11 *P*=1.000	**0.03022 ± 0.00286**		
**Treatment B OD_{600} 0.01**	1.48 *P*=0.573	1.90 *P*=0.082 °	1.16 *P*=0.997	1.27 *P*=0.948	1.00 *P*=1.000	1.04 *P*=1.000	0.93 *P*=1.000	**0.3236 ± 0.00253**	
**Axenic culture**	1.11 *P*=1.000	1.43 *P*=0.675	0.87 *P*=0.998	0.95 *P*=1.000	0.75 *P*=0.750	0.78 *P*=0.939	0.70 *P*=0.693	0.75 *P*=0.869	**0.04306 ± 0.00560**

° represents a significant difference at $P < 0.10$

Table S5. Expression levels of *AflC* from *Aspergillus flavus* and *BcSAS1* from *Botrytis cinerea* over time in *in vitro* cultures of the two fungi with living (Treatment A) or lysed (Treatment B) *Escherichia coli* HT115(DE3) bacteria producing dsRNAs against *AflC* (HT115/$AflC_{Con}$) or *BcSAS1* (HT115/$BcSAS1_{Ind}$), respectively. The expression levels of *AflC* and *BcSAS1* are shown relative to their expression when the two fungi are co-incubated with control HT115(DE3) bacteria producing dsRNAs against *eGFP* (HT115/$eGFP_{Con}$ and HT115/$eGFP_{Ind}$; respectively). Living bacteria or their whole-cell autolysates, obtained from the OD_{600} 1.0 culture, were mixed with the fungal cultures and the expression levels of *AflC* or *BcSAS*1 were measured after 12h, 24h, and 36h of co-incubation. Standard deviations (SD; ±) were obtained from three biological replicates. Significant differences between control and test applications were determined by a one-way ANOVA followed by a Tukey's HSD test, and *P* values are reported.

**Treatment - Target gene**	**12h**	**24h**	**36h**
**Treatment A – *AflC***	0.046 ± 0.005	0.192 ± 0.059	0.220 ± 0.181
(i.e. dsRNAs against *AflC)*	*P*=0.003 **	*P*=0.032 *	*P*=0.080 °
**Treatment A - *BcSAS1***	0.043 ± 0.019	0.203 ± 0.154	0.066 ± 0.020
(i.e. dsRNAs against *BcSAS1*)	*P*=0.001 **	*P*=0.099 °	*P*=0.002 **
**Treatment B – *AflC***	0.246 ± 0.163	0.591 ± 0.198	0.392 ± 0.158
(i.e. dsRNAs against *AflC*)	*P*=0.002 **	*P*=0.269	*P*=0.193
**Treatment B – *BcSAS1***	0.050 ± 0.045	0.348 ± 0.031	0.034 ± 0.027
(i.e. dsRNAs against *BcSAS1*)	*P*=0.004 **	*P*=0.096 °	*P*=0.001 **

° represents a significant difference at $P < 0.10$; * represents a significant difference at $P < 0.05$; ** represents a significant difference at $P < 0.01$.

Table S6. Differences in the expression levels of *AflC* from *Aspergillus flavus* over time among *in vitro* cultures of the fungus with living (Treatment A) or lysed (Treatment B) *Escherichia coli* HT115(DE3) bacteria producing dsRNAs against *eGFP* (HT115/$eGFP_{Con}$; control applications). Living bacteria or their whole-cell autolysates, obtained from the OD_{600} 1.0 culture, were added to the fungal cultures and the expression of *AflC* was measured at 12h, 24h, and 36h of co-incubation. Values in bold in the diagonal cells of the table represent the absolute expression of *AflC* in the co-cultures of the fungus with the living bacteria or their whole-cell autolysates. Standard deviations (SD; ±) were obtained from three biological replicates. Values in cells in the lower triangular part of the table indicate the fold-change in the expression levels of in the rows as compared to its expression levels in the matching column. Significant differences in the expression levels of *AflC* between rows and columns were determined by a one-way ANOVA followed by a Tukey's HSD test, and *P* values are reported.

	**Treatment A 12h**	**Treatment A 24h**	**Treatment A 36h**	**Treatment B 12h**	**Treatment B 24h**	**Treatment B 36h**
**Treatment A 12h**	**0.01203 ± 0.00084**					
**Treatment A 24h**	5.19, *P*=0.046 *	**0.00232 ± 0.00047**				
**Treatment A 36h**	2.54, *P*=0.093 °	0.48, *P*=0.965	**0.00473 ± 0.00074**			
**Treatment B 12h**	0.87, *P*=0.994	0.17, *P*=0.028 *	0.34, *P*=0.069 °	**0.01382 ± 0.00144**		
**Treatment B 24h**	16.81, *P*=0.012 *	3.24, *P*=0.873	6.62, *P*=0.455	19.30, *P*=0.005 **	**0.00072 ± 0.00013**	
**Treatment B 36h**	6.88, *P*=0.045 *	1.33, *P*=0.999	2.71, *P*=0.931	7.91, *P*=0.023 *	0.41, *P*=0.924	**0.00175 ± 0.00029**

° represents a significant difference at $P < 0.10$; * represents a significant difference at $P < 0.05$; ** represents a significant difference at $P < 0.01$.

Table S7. Amount of aflatoxins produced by *Aspergillus flavus* over time in *in vitro* cultures of the fungus with living (Treatment A) or lysed (Treatment B) *Escherichia coli* HT115(DE3) bacteria producing dsRNAs against *AflC* (HT115/$AflC_{Con}$; test application) or *eGFP* (HT115/$eGFP_{Con}$; control application). Living bacteria or their whole-cell autolysates, obtained from the OD_{600} 1.0 culture, were mixed with the fungal cultures and the amount of aflatoxins in the co-cultures was measured with ELISA after 12h, 24h, and 36h. Standard deviations (SD; ±) were obtained from three biological replicates. Significant differences between control and test applications were determined by a one-way ANOVA followed by a Tukey's HSD test, and *P* values are reported.

**Treatment - Application**	**12h**	**24h**	**36h**
**Treatment A - control**	15 ± 9 ppb	220 ± 38 ppb	636 ± 144 ppb
(i.e. dsRNAs against *GFP*)	n/a	n/a	n/a
**Treatment A - test**	17 ± 10 ppb	153 ± 32 ppb	225 ± 34 ppb
(i.e. dsRNAs against *AflC*)	*P*=0.858	*P*=0.082 °	*P*=0.009 **
**Treatment B - control**	17 ± 9 ppb	322 ± 61 ppb	815 ± 141 ppb
(i.e. dsRNAs against *GFP*)	n/a	n/a	n/a
**Treatment B - test**	17 ± 9 ppb	208 ± 26 ppb	470 ± 47 ppb
(i.e. dsRNAs against *AflC*)	*P*=0.979	*P*=0.041*	*P*=0.016 *

° represents a significant difference at $P < 0.10$; * represents a significant difference at $P < 0.05$; ** represents a significant difference at $P < 0.01$.

Table S8. Differences in the expression levels of *BcSAS1* from *Botrytis cinerea* over time among *in vitro* cultures of the fungus with living (Treatment A) or lysed (Treatment B) *Escherichia coli* HT115(DE3) bacteria producing dsRNAs against *eGFP* (HT115/$eGFP_{Ind}$). Living bacteria or their whole-cell autolysates, obtained from the OD_{600} 1.0 culture, were added to the fungal cultures and the expression of *BcSAS1* was measured at 12h, 24h, and 36h of co-incubation. Values in bold in the diagonal cells of the table represent the absolute expression of *BcSAS1* in the co-cultures of the fungus with the living bacteria or their whole-cell autolysates. Standard deviations (SD; ±) were obtained from three biological replicates. Values in cells in the lower triangular part of the table indicate the fold-change in the expression levels of *BcSAS1* in the rows as compared to its expression levels in the matching column. Significant differences in the expression levels of *BcSAS1* between rows and columns were determined by a one-way ANOVA followed by a Tukey's HSD test, and *P* values are reported.

	**Treatment A 12h**	**Treatment A 24h**	**Treatment A 36h**	**Treatment B 12h**	**Treatment B 24h**	**Treatment B 36h**
**Treatment A 12h**	**0.04799 ± 0.00838**					
**Treatment A 24h**	0.94, *P*=1.000	**0.05085 ± 0.01455**				
**Treatment A 36h**	0.86, *P*=0.986	0.91, *P*=0.999	**0.05564 ± 0.01038**			
**Treatment B 12h**	1.48, *P*=0.554	1.58, *P*=0.422	1.73, *P*=0.251	**0.03224 ± 0.00535**		
**Treatment B 24h**	1.27, *P*=0.893	1.35, *P*=0.785	1.48, *P*=0.567	0.86, *P*=0.984	**0.03759 ± 0.00453**	
**Treatment B 36h**	0.87, *P*=0.988	0.92, *P*=0.999	1.01, *P*=1.000	0.58, *P*=0.261	0.68, *P*=0.582	**0.05530 ± 0.00680**

Table S9. Amount of fungal biomass generated by *Botrytis cinerea* over time in *in vitro* cultures of the fungus with living (Treatment A) or lysed (Treatment B) *Escherichia coli* HT115(DE3) bacteria producing dsRNAs against *BcSAS1* (HT115/$BcSAS1_{Ind}$; test application) or *GFP* (HT115/$eGFP_{Ind}$; control application). Living bacteria or their whole-cell autolysates, obtained from the OD_{600} 1.0 culture, were mixed with the fungal cultures and the amount of fungal biomass generated in the cultures was measured in dry weight after 12h, 24h, and 36h. Standard deviations (SD; ±) were obtained from three biological replicates. Significant differences between control and test applications were determined by a one-way ANOVA followed by a Tukey's HSD test, and *P* values are reported.

Treatment - Application	12h	24h	36h
**Treatment A - control**	106 ± 8 mg	270 ± 36 mg	961 ± 223 mg
(i.e. dsRNAs against *GFP*)	n/a	n/a	n/a
**Treatment A - test**	89 ± 6 mg	196 ± 11 mg	729 ± 173 mg
(i.e. dsRNAs against *BcSAS1*)	*P*=0.045 *	*P*=0.027 *	*P*=0.230
**Treatment B - control**	166 ± 41 mg	463 ± 40 mg	1157 ± 55 mg
(i.e. dsRNAs against *GFP*)	n/a	n/a	n/a
**Treatment B - test**	101 ± 17 mg	179 ± 22 mg	878 ± 128 mg
(i.e. dsRNAs against *BcSAS1*)	*P*=0.065 °	*P*=0.001 ***	*P*=0.026 *

° represents a significant difference at $P < 0.10$; * represents a significant difference at $P < 0.05$; *** represents a significant difference at $P < 0.001$.

Table S10. Expression levels of *AflC* from *Aspergillus flavus* and the amount of aflatoxins produced by the fungus on maize kernels, following its exposure to living (Treatment A) or lysed (Treatment B) *Escherichia coli* HT115(DE3) bacteria producing dsRNAs against *AflC* (HT115/*AflC*$_{Con}$; test application) or *eGFP* (HT115/*eGFP*$_{Con}$; control application). *AflC* expression levels in the test applications are relative to the control ones (set to 1.0). *Aspergillus flavus* was spot inoculated on maize kernels and 48h later 10 μl of living bacteria or their whole-cell autolysates, obtained from the OD_{600} 1.0 culture, was applied to the same inoculation spots. Measurements were taken 24h later and standard deviations (SD; ±) were obtained from three biological replicates. Significant differences were determined by a one-way ANOVA followed by a Tukey's HSD test, and *P* values are reported.

	***AflC* relative expression**	**Aflatoxins content (ppb)**
**Treatment A / Control application (*GFP*)**	n/a	8,453 ± 2,114<br>n/a
**Treatment A / Test application (*AflC*)**	0.210 ± 0.123<br>*P*=0.021 *	4,447 ± 1,641<br>*P*=0.060 °
**Treatment B / Control application (*GFP*)**	n/a	7,872 ± 1,863<br>n/a
**Treatment B / Test application (*AflC*)**	0.153 ± 0.126<br>*P*= 0.024 *	3,791 ± 1499<br>*P*= 0.042*

° represents a significant difference at *P*< 0.10; * represents a significant difference at *P*< 0.05

Table S11. Expression levels of *BcSAS1* from *Botrytis cinerea* and size of infection areas produced by the fungus on leaves of *Nicotiana benthamiana*, following its exposure to living (Treatment A) or lysed (Treatment B) *Escherichia coli* HT115(DE3) bacteria producing dsRNAs against *BcSAS1* (HT115/$BcSAS1_{Ind}$; test application) or *eGFP* (HT115/$eGFP_{Ind}$; control application). *BcSAS1*expression levels and size of the infection areas in the test applications are relative to the control ones (set to 1.0 and 100%, respectively). *Botrytis cinerea* was spot inoculated on *N. benthamiana* leaves and 6h later 10 µl of living bacteria or their whole-cell autolysates, obtained from the OD_{600} 1.0 culture, was applied to the same inoculation spots. Measurements were taken 72h later and standard deviations (SD; ±) were obtained from four biological replicates. Significant differences were determined by a one-way ANOVA followed by a Tukey's HSD test, and *P* values are reported. Significant differences in the infection arrears between control and test applications were examined by a one-sample *t*-test.

	***BcSAS1* relative expression**	**% infection area**
**Treatment A / Test application (*BcSAS1*)**	0.338 ± 0.046<br>P= 0.001 **	64.8 ± 12.5<br>P< 0.011 *
**Treatment B / Test application (*AflC*)**	0.559 ± 0.081<br>P= 0.011 *	74.6 ± 12.6<br>P< 0.028 *

* represents a significant difference at P< 0.05; ** represents a significant difference at P< 0.01.
